# Swine inflammation and necrosis syndrome outbreak in Brazil: a case report

**DOI:** 10.1007/s11259-026-11123-5

**Published:** 2026-02-28

**Authors:** Júlia Berro, Karoline Silveira Sperb, Gabriel Pola, Paola Reis Freimuth, Brendha Lauren Fetter, Daniela Teresa Schuh, Douglas Mikael Ribeiro da Rosa, Roberta Cristina Scheid, Karine Ludwig Takeuti

**Affiliations:** 1 Universidade Feevale, Novo Hamburgo, 93525-075 RS Brazil; 2 Cooperativa Ouro do Sul, Harmonia, RS Brazil

**Keywords:** Feed, Mycotoxins, SINS, Swine

## Abstract

The outbreak occurred in three farrowing rooms from a commercial farrow-to-wean farm with 4,600 sows located in southern Brazil. During the first week of outbreak, diarrhea and vomiting were recorded in sows, and inflammatory lesions were reported in neonatal piglets. Lesions were located on the tail, hock, perineal area, teats, vulva, male genital organs, sole, coronary band, ear, and jaw, which progressed to necrosis within few days. A total of 562 sows were evaluated for the presence of diarrhea and vomiting. Their litters, comprising 8,344 suckling piglets, were individually assessed for the presence, location and type of lesions. Laboratory analysis confirmed the presence of ergosterol in feed, which indicates a poor microbiological stability and fungal yeast invasion of the feed. Diarrhea was observed in 2.3% of sows. Overall, 38.2% (*n* = 3,190) of piglets presented lesions. In farrowing unit A, the tail and claw were the most frequently affected areas, whereas sole lesions predominated in room B and teat lesions in room C. Additionally, 12.0% of piglets born from sows with high genetic potential were not selected for replacement due to teats necrosis. This is the first case report of Swine Inflammation and Necrosis Syndrome (SINS) described outside Europe. Moreover, it highlights the importance of early diagnosis and feed monitoring for the prevention of mycotoxicosis in swine, to reduce their impacts on animal welfare, gilt selection for replacement, and removal of contaminated feed from the farm.

## Background

The Swine Inflammation and Necrosis Syndrome (SINS) is a condition that combines clinical signs of inflammation and necrotic tissue in acral areas. It particularly affects the tail base and/or tip, ears, coronary bands, heels, sole, claw wall, teats, vulva, umbilicus, and face (Gerhards et al. 2025a, b; Kuehling et al. 2021b; Loewenstein et al. 2022; Reiner et al. 2019). This syndrome may be observed in suckling piglets with clinical signs being most evident during the first week of life, weaned and fattening pigs (Reiner et al. 2019, 2020). Symptomatology generally begins with bristle loss, followed by swelling and redness, and in advanced stages, exudation, and necrosis (Reiner et al. 2019, 2021). SINS as a primary endogenous disease has been associated to mycotoxins in feed, such as: zearalenone, deoxynivalenol, T-2, and ergot alkaloids (Reiner et al. 2020). Other risk factors for the occurrence of SINS are thermoregulation and inappropriate water supply, stress, genetics, and inflammatory response to lipopolysaccharides (mainly from *Escherichia coli*) (Gerhards et al. 2025a; Kuehling et al. 2021a; Reiner et al. 2019). Animals affected by SINS may experience pain and discomfort due to vasculitis and thrombosis of the vessels, decreasing the animal welfare (Gerhards et al. 2025b). The present case report describes the first time an outbreak of SINS outside Europe and the associated severe lesions in suckling piglets.

## Case presentation

The outbreak occurred in a farrow-to-wean farm with 4,600 sows, located in Rio Grande do Sul, southern Brazil, which had five farrowing rooms. Births occurred weekly in each room and piglets were weaned at 21 days of age. Housing conditions complied with recommended standards for large-scale pig production systems. Rooms were equipped with curtains and exhaust fans to ensure adequate air exchange. Each farrowing room contained approximately 200 farrowing pens with iron slatted floors and a creep area for piglets. Feed was provided through automatic feeders, and water was supplied ad libitum through nipple drinkers. The sow feed diet was based on ground corn, soybean meal, bone and meat meal, vegetable oil, limestone, salt, amino acids (lysine, methionine, and threonine), mineral premix and vitamins.

**Fig. 1:**
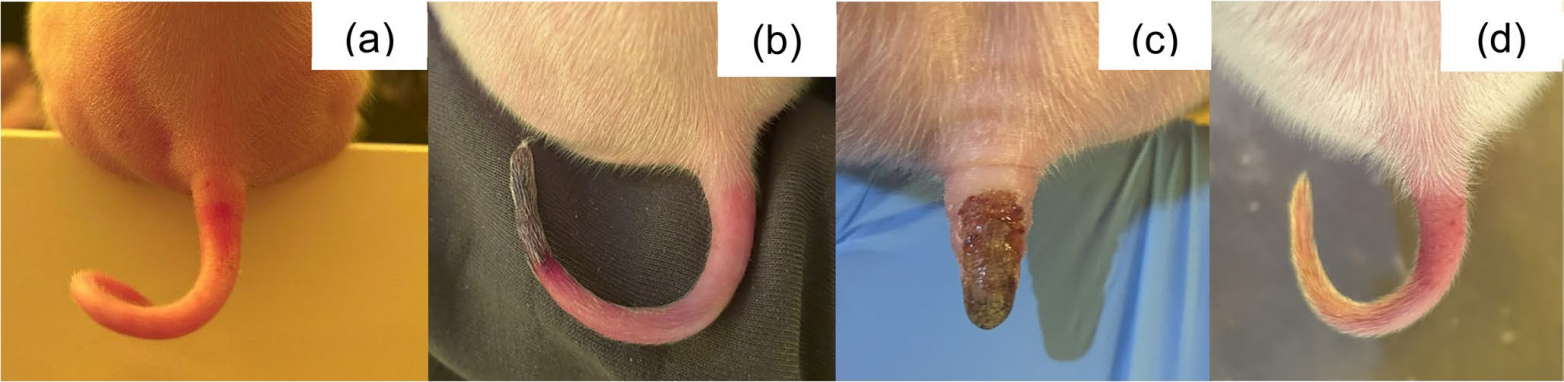
SINS lesions in the tail of suckling piglets. (**a**) Inflammation; (**b**) necrosis and inflammation; (**c**) necrosis; (**d**) scaling

**Fig. 2:**
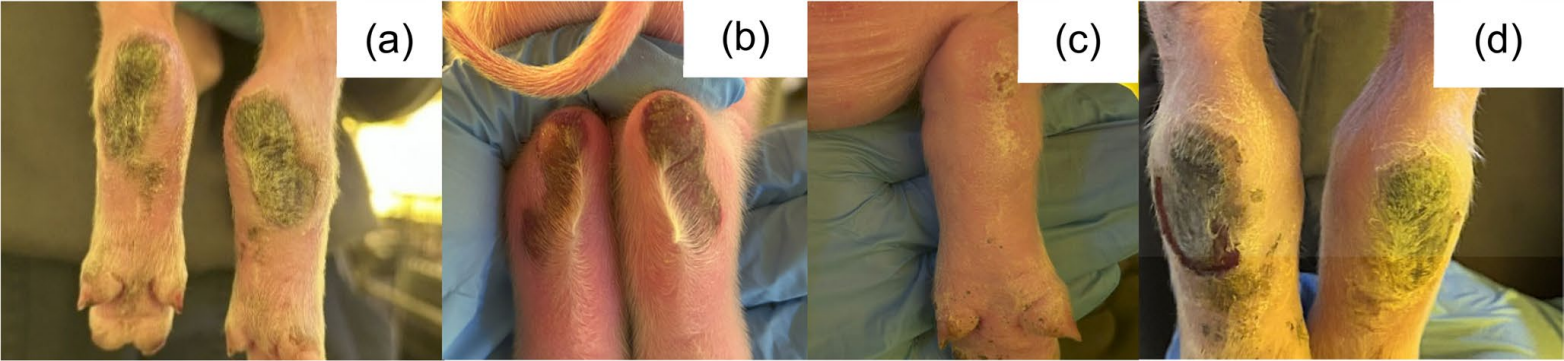
SINS lesions in the hock of suckling piglets. (**a**, **b**) necrosis; (**c**) scaling; (**d**) necrosis and injury

**Fig. 3:**
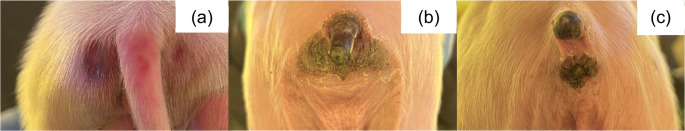
SINS lesions in the perineal area and tail of suckling piglets. (**a**) inflammation; (**b**, **c**) necrosis

**Fig. 4:**
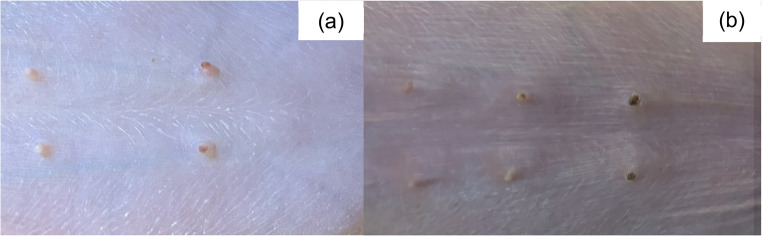
SINS lesions in teats of suckling piglets. (**a**) inflammation; (**b**) necrosis

**Fig. 5:**
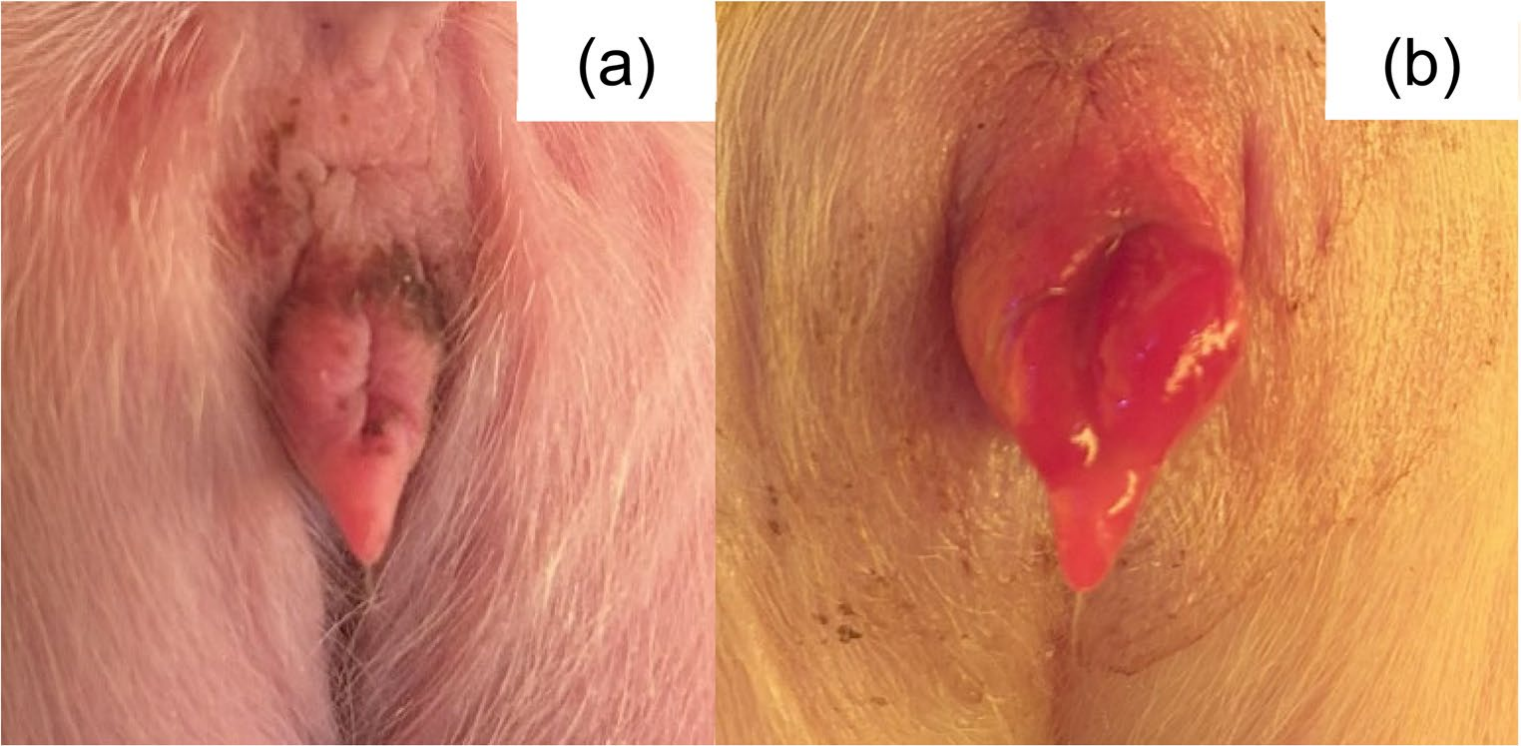
SINS lesions in the vulva of suckling piglets. (**a**) necrosis; (**b**) edema

**Fig. 6:**
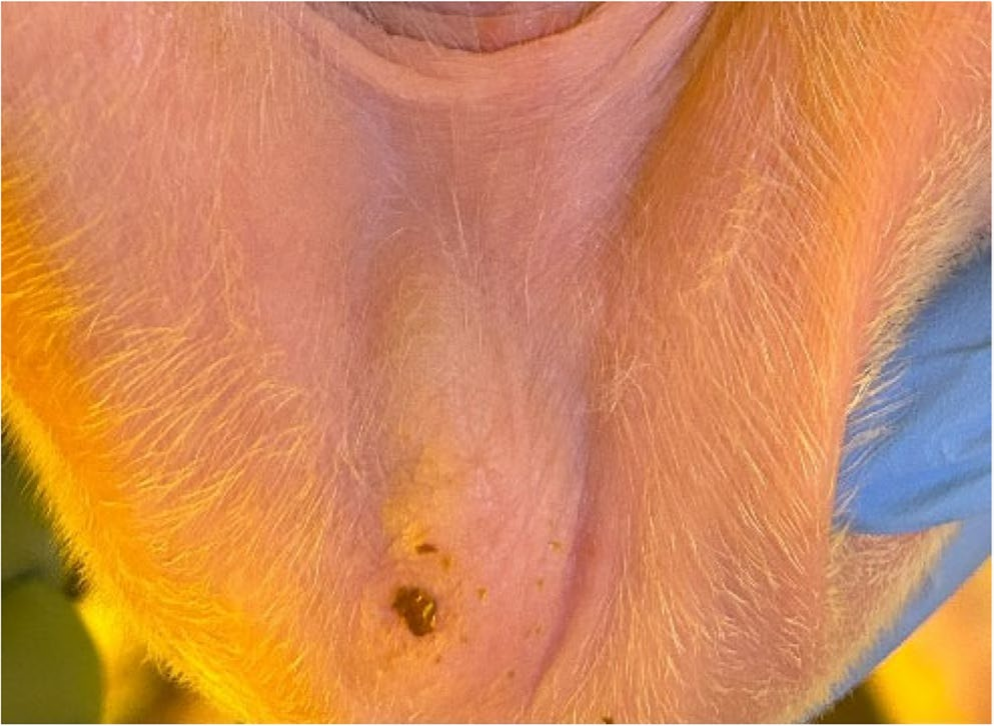
SINS lesion of necrosis in the scrotal sac of a suckling piglet

**Fig. 7:**
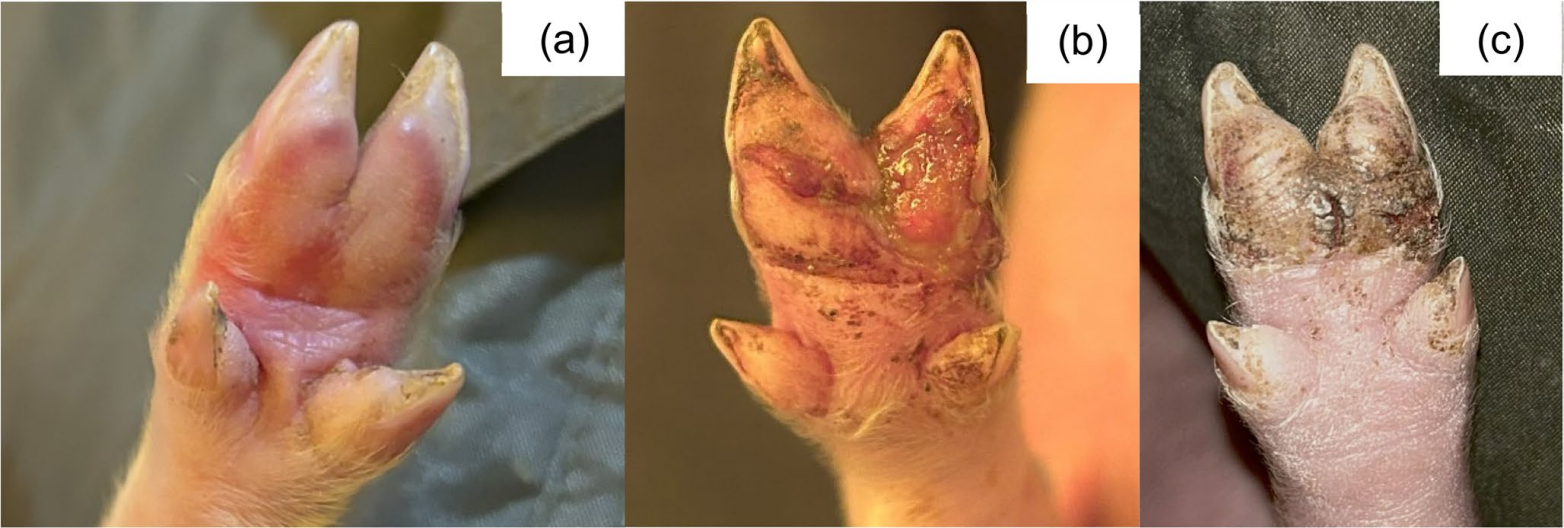
SINS lesions on the sole of suckling piglets. (**a**) inflammation; (**b**) injury; (**c**) injury and necrosis

**Fig. 8:**
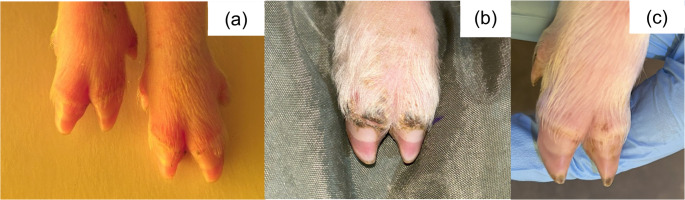
SINS lesions on the coronary band of suckling piglets. (**a**) inflammation; (**b**) necrosis; (**c**) scaling

**Fig. 9:**
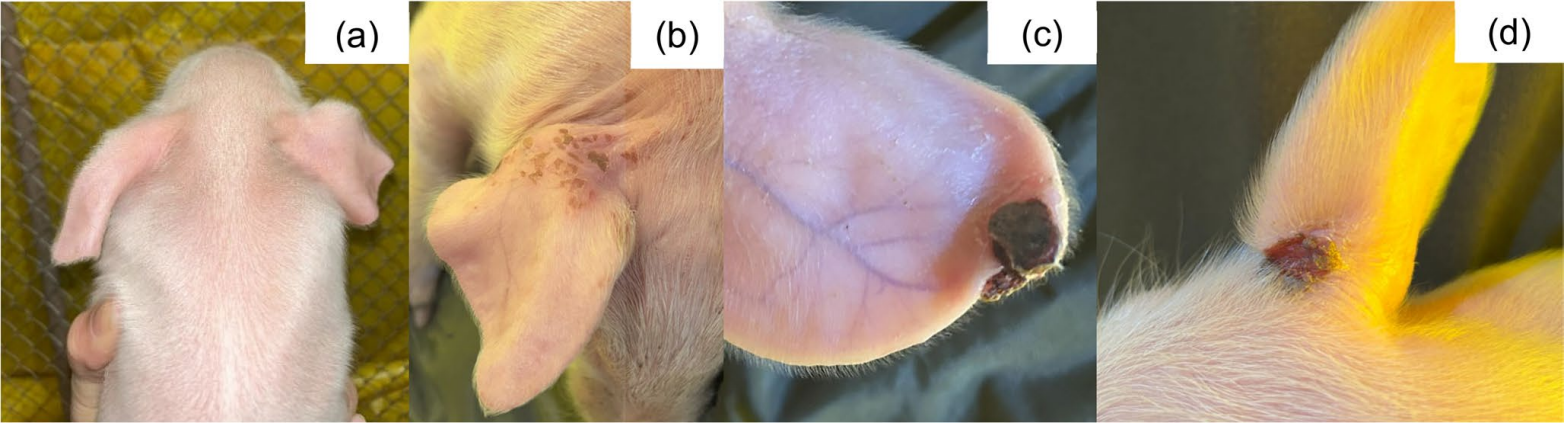
SINS lesions on the ear of suckling piglets. (**a**) edema; (**b**) scaling; (**c** and **d**) necrosis

**Fig. 10:**
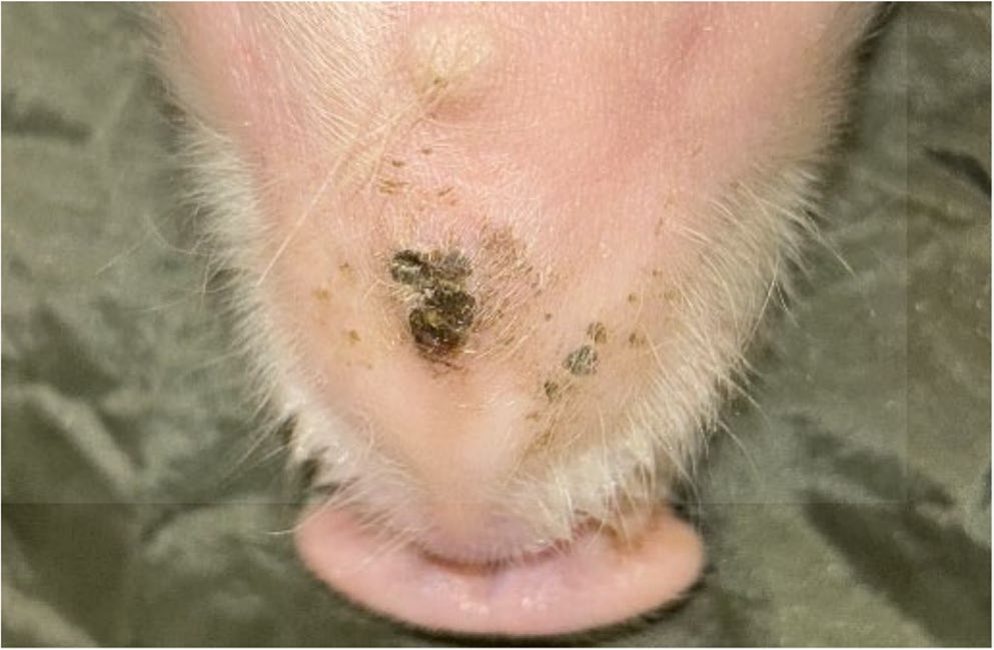
SINS lesion of necrosis in the jaw of a suckling piglet

In July 2025 (Brazilian winter), farm employees reported that sows in one farrowing room (Farrowing Room A) showed diarrhea and vomiting, and their piglets showed tail, ear, and claw inflammation in the first days of life, which progressed to necrosis within few days. The macroscopic lesions of 562 litters, corresponding to 8,344 piglets were assessed for three weeks (from birth to weaning) in three farrowing rooms (Farrowing Room A, B and C). The clinical signs of sows from the three rooms were classified according to absence or presence of diarrhea and/or vomiting. The macroscopic lesions of piglets from Farrowing Room A, which showed the most severe cases, were classified (Figs. 1, 2, 3, 4, 5, 6, 7, 8, 9 and 10) into one of the following types of lesions: inflammation, necrosis, scaling, edema, and injury (adapted from the classification of Gerhards et al. 2025a). For this classification, it was considered: (a) inflammation: characterized by redness, heat, pain and/or loss function; (b) necrosis: grayish or yellowish lesion, distinguished from the surrounding regular tissue, and presents sharply demarcated borders; (c) scaling: lesion results from accumulation of corneocytes in the stratum corneum; (d) edema: characterized by swollen and cold to the touch; (e) injury: a macroscopic alteration that affects the tissue as an open wound.

Additionally, the presence and location of lesions were assessed in Farrowing Room B and C. Feed sampling was carried out directly from the feeder in two farrowing rooms (A and B) and stored in plastic bags at room temperature for laboratory analysis to detect zearalenone and ergosterol. The analysis was carried out using High-Performance Liquid Chromatography (HPLC), with a reference value for swine of 100 µg/kg, and 20 µg/kg for ergosterol (Schwadorf and Müller 1989) and zearalenone (Kordic et al. 1992), respectively.

**Fig. 11:**
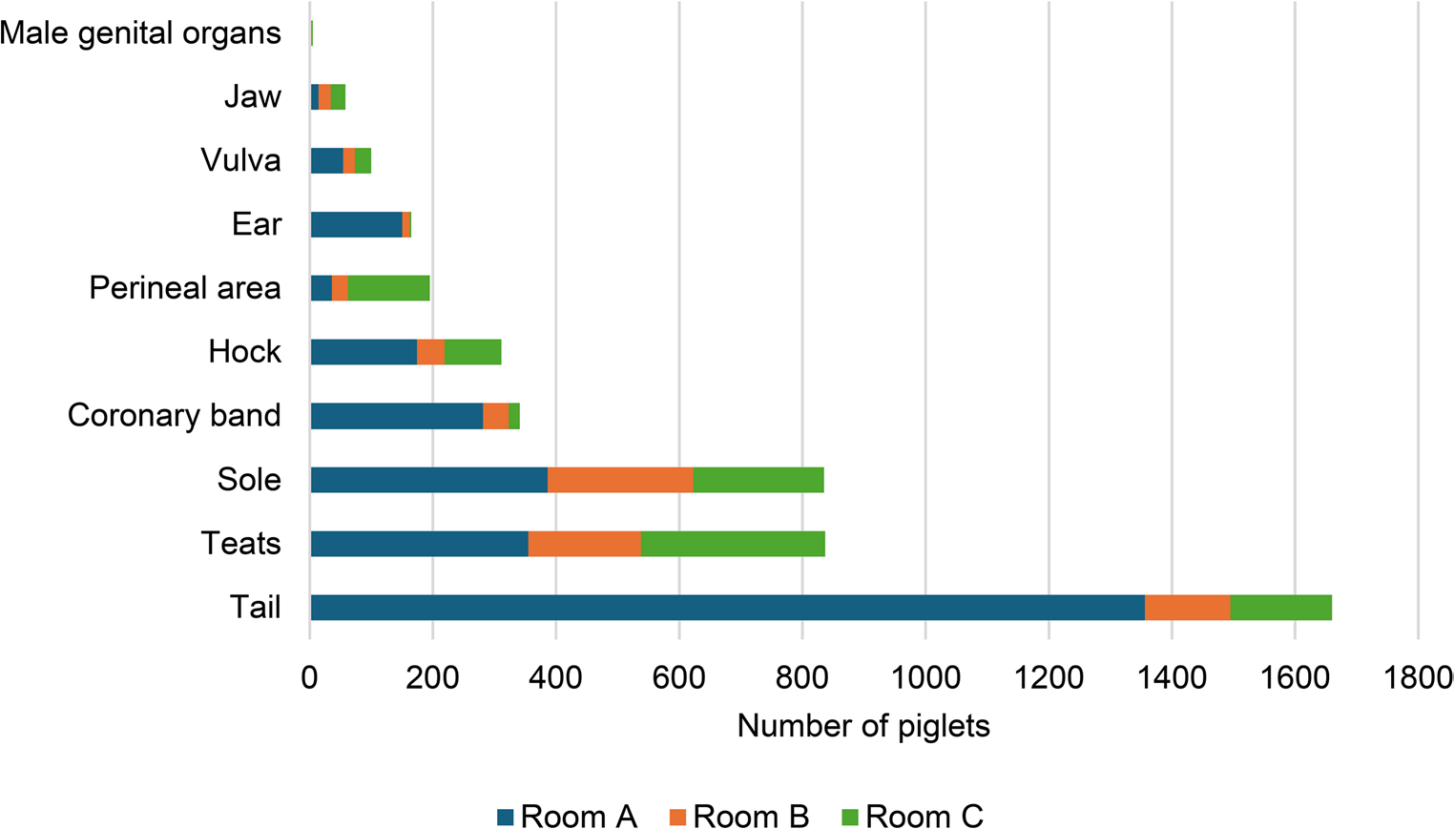
Number of suckling piglets affected by SINS regarding the lesion location in each farrowing room

A total of 4.8% (9/187), 1.6% (3/186), and 0.5% (1/189) of sows presented diarrhea in Farrowing Room A, B, and C, respectively. No signs of vomiting were detected during data collection. Piglets’ lesions were observed in the tail, hock, perineal area, teat, vulva, male genital organs (testicle and scrotum), sole, coronary band, ear, and jaw. Data from macroscopic lesions assessed in all farrowing rooms are presented in Fig. 11. A total of 38.2% of piglets (*n* = 3,190) presented lesions compatible with SINS across the three rooms. The distribution of those lesions per location was: tail (52.0%; *n* = 1,660), teat (26.2%; *n* = 837), sole (26.2%; *n* = 835), coronary band (10.7%; *n* = 341), hock (9.7%; *n* = 311), perineal area (6.1%; *n* = 195), ear (5.2%; *n* = 165), vulva (3.1%; *n* = 100), jaw (1.8%; *n* = 58) and male genital organ (0.2%; *n* = 5). In Farrowing Room A, a higher prevalence of macroscopic lesions was observed (69.1%; *n* = 1,826/2,643) compared to rooms B (18.4%; *n* = 504/2,737) and C (23.5%, *n* = 696/2,964). The most prevalent lesions were tail, sole, and teats in Farrowing Room A, sole in room B, and teats in room C. In addition, the classification of lesions in piglets from Farrowing Room A revealed that the most frequent lesion was necrosis, representing 67.6% (*n* = 1,787) of the cases, followed by inflammation (43.9%; *n* = 802). Scaling, edema and injury represented a total of 15.5% (*n* = 441).

**Fig. 12:**
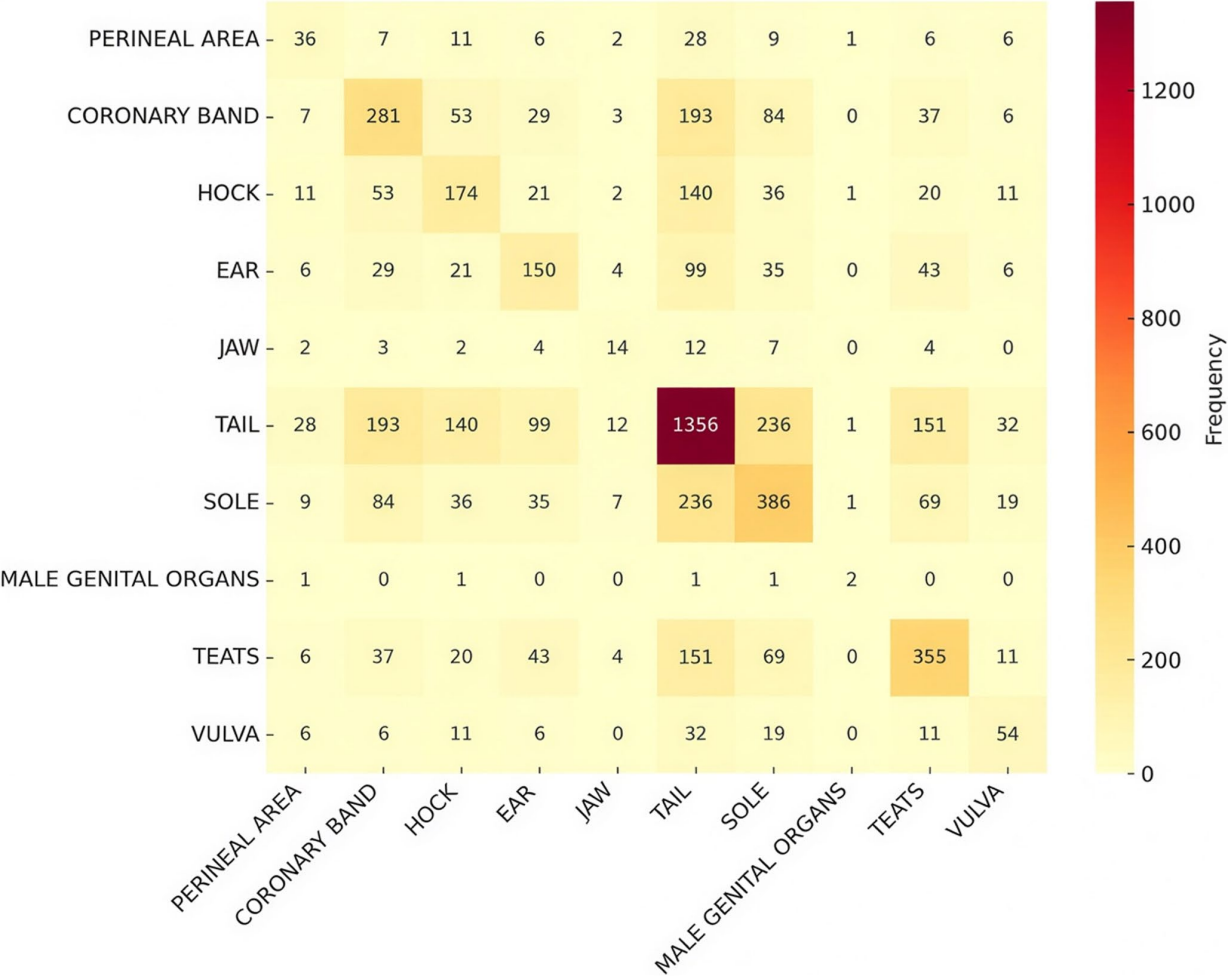
Co-occurrence of affected locations with lesions caused by SINS in suckling piglets from Farrowing Room A

**Fig. 13:**
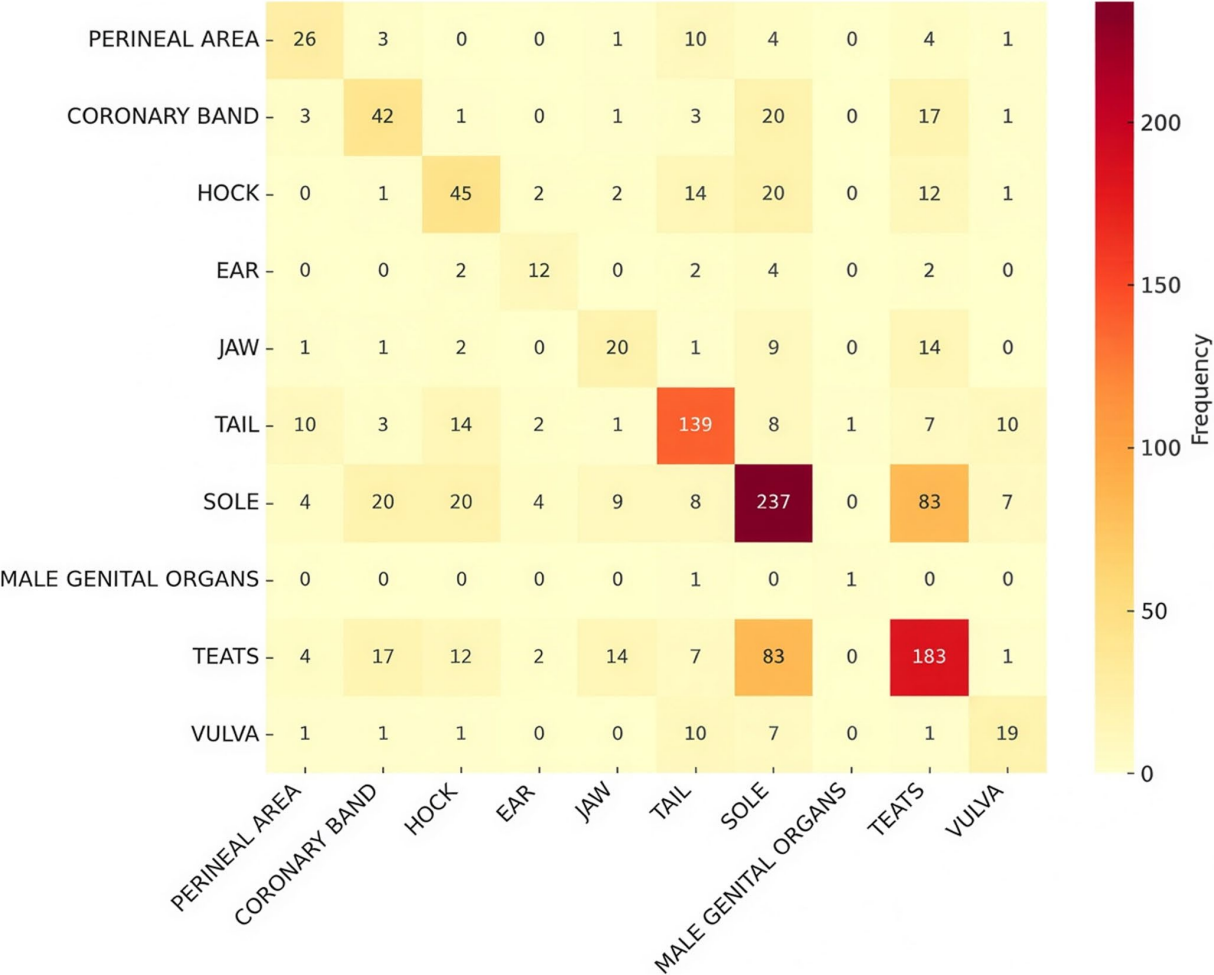
Co-occurrence of affected locations with lesions caused by SINS in suckling piglets from Farrowing Room B

**Fig. 14:**
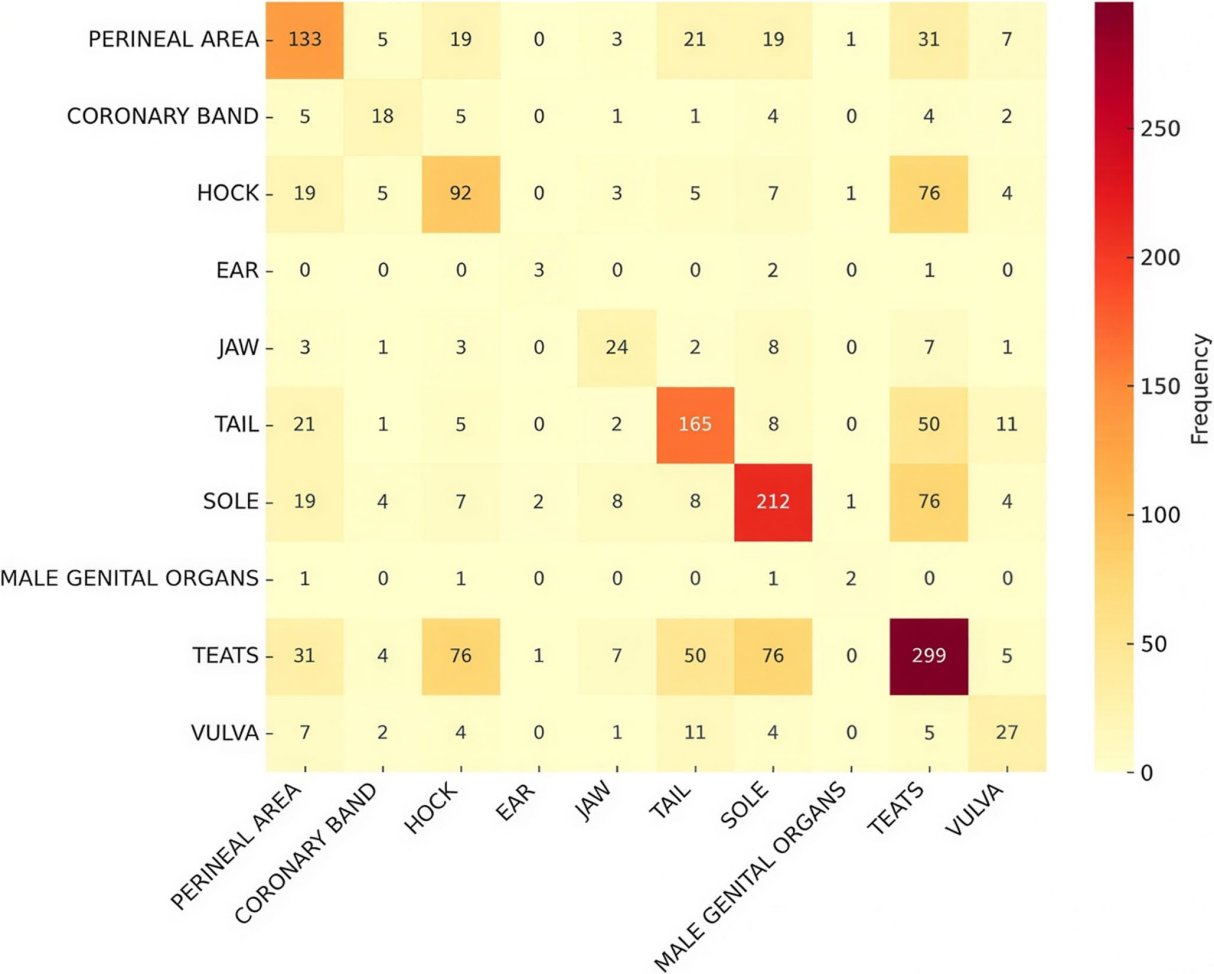
Co-occurrence of affected locations with lesions caused by SINS in suckling piglets from Farrowing Room C

The analysis of co-occurrence for each farrowing room is presented in Figs. 12, 13 and 14, highlighting the most frequent lesions that occurred in the same animal in each room. Tail and sole (12.9%), tail and coronary band (10.6%), and tail and teats (8.3%) were the most frequent lesions found simultaneously in piglets from Farrowing Room A. Concomitant lesions of teats and sole (16,5%) were observed in Farrowing Room B, whereas teats and hock (10,9%) and teats and sole (10,9%) were the most prevalent in Farrowing Room C.

Besides the commercial sows, a proportion of grandparent and great-grandparent sows were also housed in the three rooms affected. The number of sows and piglets presenting teat lesions are shown in Table 1. Overall, a total of 49.2% of sows had litters with teat lesions, resulting in 117 (12.0%) piglets that were not selected for replacement in the farm.

**Table 1:** Data from grandparent sows and great-grandparent sows whose litters were affected during the SINS outbreak. Piglets born from those sows were not selected as replacement gilts due to teat lesions

Farrowing Room	Number of grandparent and great-grandparent sows housed	Percentage of grandparent and great-grandparent sows with piglets with teat lesions	Number of total live born piglets	Percentage of piglets with teat lesion up to weaning
A	19	73.7% (*n* = 14)	279	24.0% (*n* = 67)
B	22	45.5% (*n* = 10)	329	10.9% (*n* = 36)
C	24	33.3% (*n* = 8)	366	3.8% (*n* = 14)
Total	65	49.2% (*n* = 32)	974	12.0% (*n* = 117)

The laboratory analysis revealed a total of 2,028.74 and 1,920.62 µg/kg for ergosterol in feed from farrowing rooms A and B, respectively, indicating a poor microbiological feed quality. However, zearalenone was not detected in feed samples.

## Discussion and conclusions

Ischemic lesions on body extremities in swine (tail base, tail tip, ears, teats, coronary bands and heels), have been associated with Swine Inflammation and Necrosis Syndrome (SINS), a recently described condition that is characterized by progressive inflammation in the acral areas. The lesions typically begin with bristle loss, followed by swelling and erythema, and in later stages, exudation and necrosis may be observed (Kuehling et al. 2021a; Reiner et al. 2020). Lesions compatible with SINS were observed in 38.2% of the evaluated piglets, and the most frequently affected areas were the tail (52.0%), followed by teat (26.2%) and sole (26.2%). Similar case reports have been described in European countries, although involving a lower number of piglets, showing the same type and distribution of lesions, with tail, teat and claw being also the main affected areas (Kuehling et al. 2021a, b; Reiner et al. 2019, 2020). According to Reiner et al. (2021), the syndrome particularly affects the tail base, tail tip, ears, coronary bands, heels, soles, claw walls, teats, navel, and face. In the present study, lesions were observed in more regions, such as hock, perineal region, male genital organs (testicles and scrotum) and jaw. Moreover, necrosis was the most significant lesion observed in our case report, representing 67.6% of piglets (*n* = 1,787) evaluated in Farrowing Room A. Interestingly, this type of lesion was not frequently reported in other studies, which described only a small proportion (1 to 10%) of piglets exhibiting necrosis, scaling, or exudation (Kuehling et al. 2021a; Kuehling et al 2021b). This suggests that the piglets evaluated in the present study developed more chronic and severe lesions compared with those reported previously, as bleeding, exudation and necrosis represent the final stages of lesion development and occur only in severe cases of SINS (Reiner et al. 2020).

According to Reiner et al. (2021), stressful factors, sow quality, and husbandry conditions, such as suboptimal water supply, inadequate thermoregulation, feed and water quality, poor ventilation, and other stressors, strongly contribute to the clinical presentation of SINS in piglets. These factors increase the translocation and systemic exposure to MAMPs (microbe-associated molecular patterns), due to excessive proliferation of the gut microbiota and permeability of the blood intestinal barrier, liver, and circulation. This triggers inflammatory responses at the intestine, liver, endothelium, and central nervous system. SINS scores in piglets born from low quality sows under poor housing conditions were high, but decreased significantly when husbandry conditions were improved. Other factors that may influence the severity of SINS include unfavorable genetic variants predisposing to the syndrome (Gerhards et al. 2025a; Leite et al. 2023), infectious agents, and anti-nutritional factors, such as mycotoxins, which are known to affect intestinal integrity and the innate immune system in the early stages of life, increasing the risk of inflammatory syndromes (Reiner et al. 2019).

The housing, food and water supply, and environmental conditions of the farm followed the high standard recommendations for large pig production systems and were constant over the years. However, two main factors may have contributed to the emergence of the outbreak: (a) it occurred during the winter, when the temperatures outside the barns ranged from 0 °C to 18 °C, with an average of 8 °C, which is out the thermoneutral zone for swine. Although temperatures inside each room were not measured, they were likely below the recommended range for pigs, since environmental control in the barns was based on fans and curtain management, which does not prevent reductions in the room temperature. This factor could have contributed to a vasoconstrictor effect (Evans 2011); (b) the sow feed was produced by the pig company following less stringent quality-control standards. Laboratory analysis of the feed revealed a high contamination with ergosterol, a common indicator of feed spoilage by fungi and yeasts. Although feed ingredients (corn and soybean meal) should be routinely tested for several mycotoxins, such as zearalenone, deoxynivalenol, fumonisin, and aflatoxin, in this case the final feed was not analyzed prior to sow feeding, and the silos were not cleaned and disinfected between refills with various feed batches. In addition, relative humidity ranged between 70% and 80%, which is highly favorable for fungal growth and mycotoxins production. The feed analysis identified indeed high amounts of ergosterol, ranging from 19.20 to 20.28 times above the expected limit, indicating a poor microbiological quality of the feed. Mycotoxins may contribute to the development of SINS through dysfunction of the gut–liver axis. These toxins can compromise intestinal barrier integrity, increasing epithelial permeability and facilitating the translocation of bacterial products or MAMPs into the circulation. The liver, which plays a central role in mycotoxin detoxification and in the clearance of gut-derived endotoxins, may have its detoxification capacity overwhelmed, thereby allowing endotoxins and inflammatory mediators to enter the systemic circulation. This cascade may lead to endothelial dysfunction and peripheral inflammation, providing a biologically plausible mechanism for the inflammatory and necrotic lesions observed in SINS (Akbari et al. 2017; Albillos et al. 2020; Triger et al. 1978; Ponziani et al. 2018; Reiner et al. 2021). As already reported by previous authors (Jadamus and Schneider 2002; Van Limbergen et al. 2017), the mechanisms underlying the occurrence of SINS may involve thermoregulatory and nutritional factors. The presence of mycotoxins and lipopolysaccharides (LPS) in sow milk directly has been associated with necrotic lesions of the tail, ear, and coronary band, which are attributed to the inflammation of blood vessels in the affected regions (Reiner et al. 2021). The environmental conditions during the outbreak, combined with the presence of multiple fungi, as indicated by the ergosterol measurements in sow feed, may have increased the severity and morbidity of SINS, especially because the prevalence of clinical signs in the sow herd and their litters decreased over time as the feed batch was consumed.

Ergosterol is usually found in fungal plasma membrane (Dupont et al. 2012) and is considered an indicator of fungal contamination in feed (Alba-Mejía et al. 2023). For this reason, it is not a reliable indicator of mycotoxin production and indeed no zearalenone was detected. The presence of other mycotoxins in the feed was not measured in the field study, which is a limitation in the etiological characterization of this case report. In swine, intoxication with ergot alkaloids is responsible for gangrenous outbreaks resulting from pronounced vasoconstriction, which compromises peripheral circulation, resulting in ischemic lesions that are frequently symmetrical, particularly in the extremities (Ayarragaray 2014; Torres et al. 2020; Zimmerman et al. 2012). Ergot alkaloids also exert oxytocic effects, leading to stimulation of the nervous system followed by depression, as well as reproductive disorders, including abortion (Zimmerman et al. 2012). On the other hand, SINS is characterized by an inflammatory process in which lesions develop in a multifocal pattern, affecting different body regions and progressing through multiple stages, ranging from early inflammatory changes to necrosis, with varying degrees of severity coexisting within the same animal (Kuehling et al. 2021a; Reiner et al. 2019, 2021). Although both conditions may present with necrosis of the extremities, the irregular distribution of lesions, together with the presence of lesions at different stages of progression and the absence of neurological and reproductive signs, supports a diagnosis of SINS and rules out ergotism as the primary cause of the observed alterations.

The economic impact of SINS is related to reduced animal welfare and may also be associated with lower piglet body weight at birth and at weaning (Leite et al. 2023). Moreover, as the breeding herd was renewed exclusively by self-replacement, litters born from grandparents and great-grandparents were assessed in farrowing rooms A, B and C. A total of 12.0% of piglets presented teat lesions, suggesting an important impact on gilts selection criteria. Every single necrotic teat must be considered an injured teat, and those gilts must be excluded from replacement selection (PIC 2023). In addition, even though foot lesions are commonly associated with poor flooring (Heimann et al. 2024), those findings were not commonly reported in the farm prior to the outbreak, suggesting that SINS could have increased their emergence, and could therefore be considered an additional criterion for replacement gilt selection.

The occurrence of SINS may directly impact on sows and suckling piglets’ welfare, colostrum and milk production/intake, culling of self-replacement gilts prior to weaning, and feed withdrawal from the farm. The outbreak reported in this study suggests a possible mixed pathogenesis of SINS, associated with environmental conditions during the outbreak and the effects of mycotoxins, which may have acted as a cofactor in the manifestation and modulation of SINS, increasing both the number of affected piglets and lesion severity. Finally, it is important to emphasize the relevance of mycotoxins control and early diagnosis, especially in countries where SINS has not been previously reported.

## Data Availability

No datasets were generated or analysed during the current study.
